# Catalysis of proline isomerization and molecular chaperone activity in a tug-of-war

**DOI:** 10.1038/s41467-020-19844-0

**Published:** 2020-11-27

**Authors:** Filippo Favretto, David Flores, Jeremy D. Baker, Timo Strohäker, Loren B. Andreas, Laura J. Blair, Stefan Becker, Markus Zweckstetter

**Affiliations:** 1 German Center for Neurodegenerative Diseases (DZNE), Von-Siebold-Str. 3a, 37075 Göttingen, Germany; 2 Department of Molecular Medicine, Morsani College of Medicine, USF Health Byrd Alzheimer’s Institute, University of South Florida, Tampa, FL 33613 USA; 3 Department for NMR-based Structural Biology, Max Planck Institute for Biophysical Chemistry, Am Faßberg 11, 37077 Göttingen, Germany

**Keywords:** Biochemistry, Intrinsically disordered proteins, Solution-state NMR

## Abstract

Catalysis of cis/trans isomerization of prolines is important for the activity and misfolding of intrinsically disordered proteins. Catalysis is achieved by peptidylprolyl isomerases, a superfamily of molecular chaperones. Here, we provide atomic insight into a tug-of-war between cis/trans isomerization and molecular chaperone activity. Catalysis of proline isomerization by cyclophilin A lowers the energy barrier for α-synuclein misfolding, while isomerase-binding to a separate, disease-associated protein region opposes aggregation. We further show that cis/trans isomerization outpowers the holding activity of cyclophilin A. Removal of the proline isomerization barrier through posttranslational truncation of α-synuclein reverses the action of the proline isomerase and turns it into a potent molecular chaperone that inhibits protein misfolding. The data reveal a conserved mechanism of dual functionality in cis/trans isomerases and define its molecular determinants acting on intrinsically disordered proteins.

## Abstract (web summary)

Cyclophilin A (CypA) is a peptidylprolyl isomerase that also has chaperone activity and interacts with the intrinsically disordered protein α-Synuclein (aSyn). Here, the authors combine NMR measurements and biochemical experiments to characterise the interplay between the catalysis of proline isomerization and molecular chaperone activity of CypA and find that both activities have opposing effects on aSyn and further show that the that cis/trans isomerization outpowers the holding activity of CypA.

## Introduction

Intrinsically disordered proteins (IDPs) and disordered regions mediate a wide range of interactions and thus contribute to diverse biological and pathological processes^1^. Besides a bias toward hydrophilic residues and amino-acid repeats, the sequences of IDPs often contain proline residues^2^. The *cis*-/*trans*-isomerization of prolyl peptide bonds is a slow reaction but can be catalyzed by peptidylprolyl isomerases (PPIases), ATP-independent molecular chaperones that bind their clients using a promiscuous interface^3–5^. In agreement with the important role of PPIases for catalysis of *cis*-/*trans*-isomerization of proline residues, PPIases have been associated with misfolding and pathogenic aggregation of IDPs^6–9^. In addition, specific *cis*-conformers of IDPs are linked to age-dependent neurodegeneration^10,11^. Because IDPs do not have to undergo a folding process, the connection between proline isomerization and molecular chaperone activity of PPIases acting on IDPs is however enigmatic.

The IDP α-synuclein (aSyn) is intimately connected to the development and progression of Parkinson’s disease and other neurodegenerative disorders^12,13^. Amyloid-like aggregates of aSyn are a major component of cytoplasmic protein/lipid inclusions in the brain of PD patients^12^. The core of aSyn amyloid aggregates is formed by the central part of the aSyn sequence^14–17^. Downstream of the aggregation-prone segments, aSyn has a negatively charged C-terminal domain that harbors five proline residues^18^. The proline residues are important for aSyn misfolding and aggregation^19^. Post-translational truncation of aSyn results in shortened aSyn variants, which contain less or no prolines and aggregate more rapidly^20^.

Recently, it was shown that aSyn binds to molecular chaperones in the cytosol of mammalian cells^21^. Inhibition of the aSyn/chaperone interaction results in the binding of aSyn to mitochondria and aSyn aggregation. One of the six chaperones that were reported to bind to aSyn in the cytosol of mammalian cells is cyclophilin A (CypA)^21^. CypA belongs to the evolutionarily conserved family of cyclophilin PPIases^22^ and makes up ∼0.1–0.6% of the total cytosolic proteins^22^. Further support for the role of *cis*-/*trans*-isomerization of prolines in neurodegeneration comes from the observation of PPIase-positive inclusions in patients with α-synucleinopathies^23^. In addition, CypA is associated with other major human diseases such as cardiovascular diseases, viral infections, rheumatoid arthritis, asthma, and cancer^24^.

Here, we provide atomic insight into the interplay between catalysis of proline isomerization and molecular chaperone activity of CypA acting on aSyn. We reveal a tug-of-war between catalysis of *cis*-/*trans*-isomerization, which lowers the energy barrier for aSyn misfolding and thus catalyzes aSyn aggregation, and a highly potent amyloid-blocking chaperone activity of CypA. The dual mechanism of catalysis of proline isomerization and molecular chaperone activity is shared by other members of the cyclophilin family.

## Results

### CypA catalyzes aSyn aggregation

**Fig. 1:**
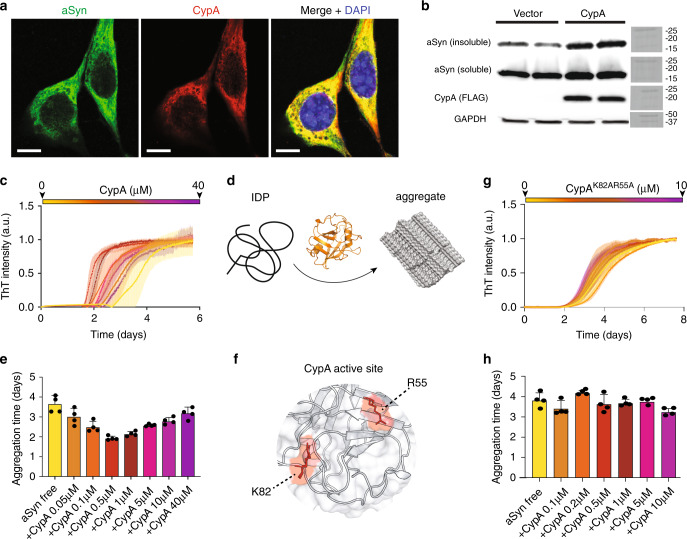
CypA catalyzes aSyn aggregation. **a** HT-22 cells transfected with CypA and aSyn and stained for aSyn (green), CypA (red), and DNA (DAPI, blue) were imaged at 63× original magnification using a confocal microscope. Scale bars represent 10 µm. The images were acquired from *n* = 10 fields of 2 independent experiments. **b**, Western blots from HEK293T cells transfected for 72 h with aSyn, as well as empty vector or CypA plasmids expressing for 72 h following Sarkosyl-insoluble fractionation. *n* = 3 independent experiments were performed**. c**, **g** ThT-fibrillization assay of aSyn alone (30 μM, green) and in the presence of increasing concentrations of wild-type CypA (**c**) or the functionally impaired R55A/K82A mutant of CypA (**g**). Data points represent the mean values from *n* = 4 independent experiments ±1 standard deviation (SD). **d** Schematic representation illustrating catalysis of IDP misfolding by CypA. The aggregate was prepared with PyMol v. 1.7.6.0 using aSyn amyloid fibrils (PDB code: 6A6B). **e**, **h** Aggregation half-times of aSyn alone (yellow), and in the presence of increasing concentrations of wild-type CypA (**e**) or the R55A/K82A mutant of CypA (**h**). Data points represent the mean values from *n* = 4 independent experiments ±1 SD. **f** 3D structures of the active site of CypA. R55 and K82, which are important for CypA activity, are highlighted in red.

CypA and aSyn are present in the cytosol of neurons^21,25^. To determine if CypA interacts with aSyn, we performed confocal imaging in HT-22 cells, a mouse hippocampal neuronal line. We observed perinuclear colocalization between the two proteins (Fig. 1a), in agreement with quantitative mass spectrometry that demonstrated a direct aSyn/CypA interaction^21^. The effect of CypA on aSyn aggregation, as measured by changes in solubility, was subsequently investigated in HEK293T cells. CypA overexpression increased Sarkosyl-insoluble aSyn levels (Fig. 1b; Supplementary Fig. 1). In addition, CypA overexpression increased soluble aSyn levels (Fig. 1b), potentially due to PPIase-induced effects on aSyn transcription/translation.

To gain insight into how CypA modulates aSyn aggregation, we performed in vitro amyloid-formation experiments (Fig. 1c). Because the cellular machinery is absent in these experiments, indirect effects due to CypA overexpression can be excluded. CypA protein in concentrations from 0.05 to 40 µM was added to 30 µM of aSyn, followed by incubation at 27 °C for 6 days (Fig. 1c). From 0.05 to 0.5 µM, the half-time of aggregation gradually decreased (Fig. 1c–e). At 0.5 µM, which corresponds to an aSyn:CypA molar ratio of 60:1, i.e., 60-fold less enzyme than the substrate, the aggregation half-time was shortened to 1.9 days when compared to 3.8 days for free aSyn (Fig. 1e). Surprisingly, however, the aggregation kinetics slowed down when the CypA concentration was further increased (Fig. 1c, e). At 40 µM, CypA corresponding to a 33% excess of CypA over aSyn, the aggregation half-time was approaching that of free aSyn (Fig. 1e).

The faster fibrillization at substoichiometric CypA concentrations suggests that the underlying mechanism is connected to the catalytic activity of CypA. To provide further support for the contribution of the PPIase isomerization activity, we prepared a double mutant of CypA, in which the two residues R55 and K82 were mutated to alanine. R55 and K82 of CypA are located on the peptide-binding surface of CypA (Fig. 1f) and are important for catalysis of CypA-mediated *cis*-/*trans*-isomerization^26^. The mutations abrogated the catalytic effect of CypA on aSyn fibrillization (Fig. 1g, h).

### CypA binds aSyn

**Fig. 2:**
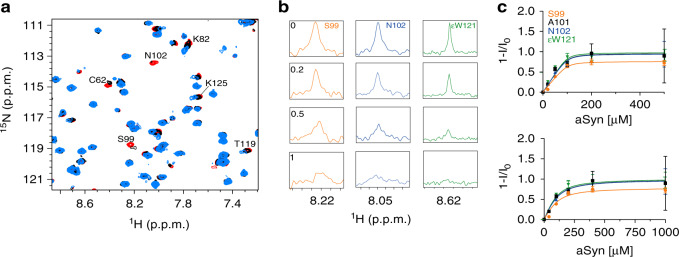
CypA binds aSyn. **a** Superposition of selected regions of ^1^H–^15^N HSQC spectra of ^15^N-labeled CypA in the presence of increasing aSyn concentrations (CypA:aSyn molar ratios of 1:0, 1:1, and 1:5 in red, black, and light blue, respectively). **b** One-dimensional ^1^H traces through selected ^1^H–^15^N cross-peaks (S99, N102, and εW121) of CypA in the presence of increasing concentrations of aSyn. aSyn:CypA molar ratios are indicated in the S99 panels. **c** Intensity changes of cross-peaks in CypA, which have predominantly slow-exchange behavior in the presence of increasing aSyn concentrations. Error bars were derived from signal-to-noise ratios in the NMR spectra of each titration experiment. Lines represent global fits to the experimental data assuming a reversible 1-to-1 binding model.

**Fig. 3:**
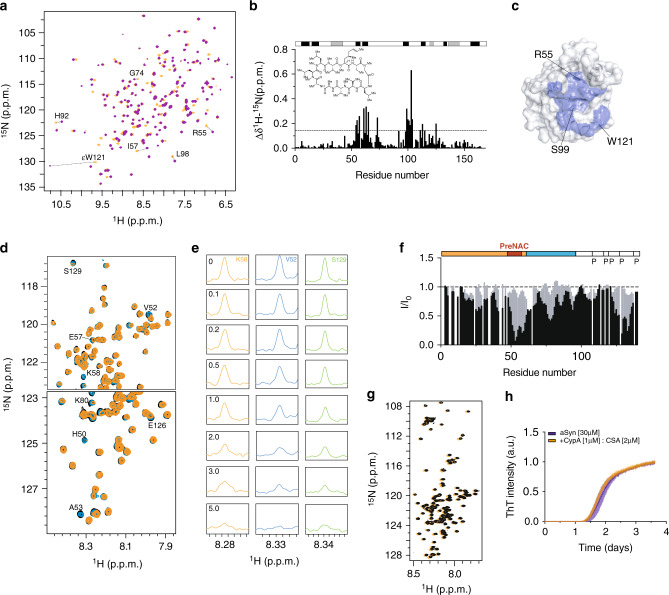
Cyclosporin A inhibits aSyn-directed CypA activity. **a** Superposition of ^1^H–^15^N HSQC spectra of CypA in the absence (orange) and presence (purple) of cyclosporin A. Selected cross-peaks are labeled by residue type and number. **b** Residue-specific chemical shift perturbation observed in (**a**). The chemical structure of cyclosporin A is shown as the inset. The location of α-helices (gray) and β-strands (black) is illustrated on top. **c** Mapping of cyclosporin A-induced chemical shift changes onto the 3D structure of CypA (PDB code: 6I42)**. d** Superposition of selected regions of ^1^H–^15^N HSQC spectra of ^15^N-labeled aSyn in the presence of increasing concentrations of CypA (CypA:aSyn molar ratios of 0:1, 1:1, and 5:1 in black, blue, and orange, respectively). **e** One-dimensional ^1^H traces through the ^1^H–^15^N cross-peaks of V52, K58, and S129 of aSyn shown in (**d**). CypA:aSyn molar ratios are indicated in the panels of K58. **f** Residue-specific attenuation of cross-peaks in ^1^H–^15^N HSQC spectra of aSyn upon addition of a fivefold molar excess of CypA (black) and CypA:CSA complex (gray). I_0_ and I are the intensities of ^1^H–^15^N HSQC cross-peaks in the absence and presence of the enzyme/complex, respectively. The domain organization of aSyn and the location of proline residues is shown on top. **g** Superposition of ^1^H–^15^N HSQC spectra of aSyn in the absence (black) and presence (orange) of the CypA/CSA complex. **h** ThT-fibrillization assay of aSyn alone (30 μM, blue) and in the presence of the CypA/CSA complex (orange). The concentrations of CypA and CSA were 1 and 2 µM, respectively. Data points represent the mean values from *n* = 4 independent experiments ±1 SD.

The interaction of CypA with aSyn was characterized using nuclear magnetic resonance (NMR) spectroscopy. Upon addition of increasing concentrations of aSyn, selected cross-peaks in two-dimensional ^1^H–^15^N HSQC spectra of CypA displayed strong signal broadening (Fig. 2a, b), but no or only very small chemical shift changes (Fig. 2a). This is a characteristic signature of a binding process that is slow to intermediate on the NMR timescale. Quantitative analysis of the aSyn-induced signal broadening of strongly attenuated, non-shifting cross-peaks (e.g., S99, A100, and N101, Fig. 2b) resulted in a *K*_D_ of 3.9 ± 2.3 μM (Fig. 2c). This is an apparent *K*_D_ value assuming a single CypA-binding site in aSyn. However, because aSyn binds with both the PreNAC stretch and the C-terminal region to CypA (see below, Fig. 3d–f), the molar concentration of aSyn-binding sites can be assumed to be twice as large as the concentration of the aSyn protein in the sample. Taking into account the two binding sites, i.e., doubling the effective aSyn concentration when fitting the NMR signal broadening, the *K*_D_ value decreased to 41.5 ± 8.5 μM. The affinity of the CypA/aSyn interaction is comparable to that of CypA binding to the HIV-1 capsid protein (~7^27^ to ~22 μM^28^).

### Cyclosporin A inhibits aSyn-directed CypA activity

The specificity of CypA activity on aSyn aggregation was further investigated by blocking CypA’s catalytic site with cyclosporin A (CSA). CSA is a powerful inhibitor of cyclophilins and is used to prevent organ rejection in people receiving transplants^29^. In addition, CSA promotes neurorestorative and cell-replacement therapy in preclinical models of Parkinson’s disease^9^.

CSA was titrated into a solution of 260 μΜ ^15^N-labeled CypA to a final CSA:CypA molar ratio of 2:1 and the CypA/CSA interaction was investigated by two-dimensional NMR spectroscopy (Fig. 3a). Comparison with the ^1^H–^15^N HSQC spectrum of CypA in the absence of CSA revealed chemical shift changes for several CypA residues (Fig. 3a). Because chemical shift changes are induced by changes in the chemical environment of amino acids, as well as changes in local dynamics and the exchange between the free and bound form of CypA, they provide insights into the binding process. Residue-specific analysis localized the chemical shift changes to the active site of CypA (Fig. 3b, c)^30^.

We then analyzed the effect of CSA on the binding of CypA to aSyn. NMR spectroscopy in combination with X-ray crystallography previously demonstrated that CypA binds independently to two sites in aSyn: the PreNAC region and the C-terminal proline-rich domain of aSyn^31^. In agreement with these data, we observed strong signal broadening for residues 49–62 and 118–131 of aSyn upon addition of increasing concentrations of CypA (Fig. 3d–f). In weaker signal attenuation was observed in aSyn’s N-terminal domain and the NAC region (Fig. 3f, black bars). In contrast, the ^1^H–^15^N HSQC of aSyn in the presence of CypA/CSA was highly similar to the spectrum of aSyn alone (Fig. 3g). The residue-specific analysis confirmed that no NMR signal broadening was present (Fig. 3f; gray bars). The inhibitor CSA thus blocks the binding of both the PreNAC region and aSyn’s C-terminal domain to the active site of CypA.

We then repeated the ThT-fibrillization assay of aSyn in the presence of 1 µM CypA, but now bound to CSA (CSA:CypA molar ratio of 2:1). While in the absence of the inhibitor, CypA strongly accelerated fibrillization of aSyn (Fig. 1c, e), the kinetics of aSyn alone, and in the presence of CSA/CypA were indistinguishable (Fig. 3h). The combined data demonstrate that the enzymatic activity of CypA plays a central role in the acceleration of aSyn fibrillization.

### Structure of aSyn’s proline-rich region bound to CypA

To gain insight into the molecular details of the CypA-induced acceleration of aSyn aggregation, we characterized the structure of CypA in complex with aSyn’s proline-rich region. In the NMR-binding study, residues 118–131 of aSyn were strongly broadened in the presence of a 5-fold excess of CypA (Fig. 3f, black bars). We, therefore, prepared an aSyn peptide comprising residues 118–131 of aSyn (termed aSyn^Ctail^) by solid-phase synthesis and performed an NMR-based interaction study of aSyn^Ctail^ with CypA (Supplementary Fig. 2). The experimentally observed perturbations of CypA cross-peaks were converted into distance restraints and used to dock aSyn^Ctail^ to CypA using the software Haddock^32^. Subsequently, the lowest-energy complex model was subjected to unrestrained Rosetta calculations^33^: 100,000 conformations of the CypA/aSyn^Ctail^ complex were calculated, clustered, and ranked by energy (Supplementary Fig. 3). For the five lowest-energy clusters, the residue-specific contacts for each conformer were analyzed and compared to the chemical shift perturbation profiles of CypA and full-length aSyn. The contact profiles of clusters 1 and 3 are in good agreement with the experimental chemical shift perturbation profiles of both proteins (Supplementary Fig. 4).

In the lowest-energy complex structure from cluster 1 (Supplementary Fig. 5), residues V118–E131 of aSyn snuggle into the CypA cleft where proline-containing peptides and CSA bind (Fig. 3c). In this conformation, P128 of aSyn, the proline residue with the strongest CypA-induced signal broadening (Fig. 3d–f), is in close proximity to the sidechain of R55 of CypA (Supplementary Fig. 5a, b). In addition, K82, which is also important for catalysis of CypA-mediated *cis*-/*trans*-isomerization^26^, is close to the two glutamates at positions 130 and 131 of aSyn (Supplementary Fig. 5a). Mutation of R55 and K82 is therefore expected to attenuate binding to aSyn and catalysis of *cis*-/*trans*- isomerization of aSyn proline residues, in agreement with the impaired ability of R55A/K82A–CypA to modulate aSyn fibrillization (Fig. 1g-h).

### CypA activity modulates aSyn amyloid structure

To probe the structure of aSyn fibrils formed in the presence of CypA (Supplementary Fig. 6a), we performed solid-state NMR experiments optimized for rigid residues located in cross-β-structure. Two-dimensional N–C and C–C correlation spectra demonstrated changes in position and intensity of some resonances when aSyn was aggregated in the presence of a substoichiometric amount of CypA (aSyn:CypA molar ratio of 1:0.17) (Supplementary Fig. 6b, c). Based on previously determined resonance assignments^34,35^, a few of the perturbed, well-separated cross-peaks were assigned to G41, S42, T59, G68, and T81 (Supplementary Fig. 6b), suggesting that CypA activity modulates the structure of aSyn fibrils.

### Overcoming a Parkinson’s disease-associated isomerization barrier

**Fig. 4:**
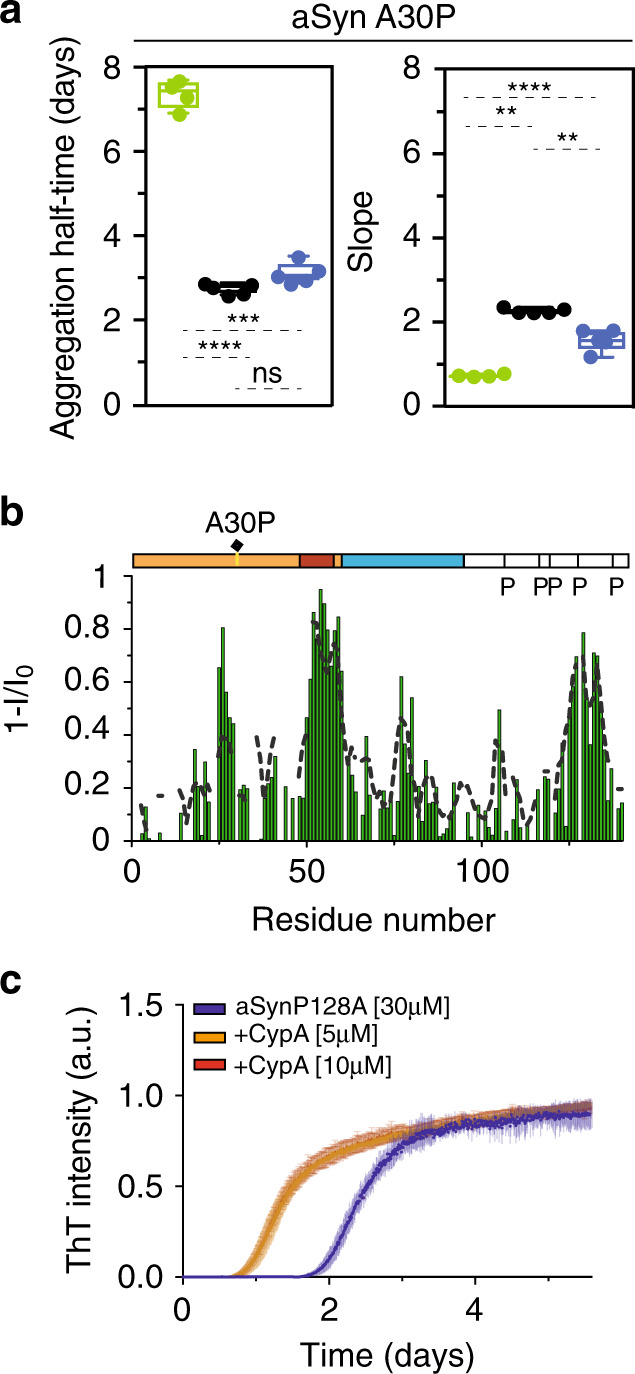
CypA overcomes the A30P Parkinson’s disease-associated isomerization barrier. **a** Aggregation kinetics of A30P aSyn in the absence (green) and presence of CypA. Data from aSyn:CypA molar ratios of 3:1 and 6:1 are shown in blue and black, respectively. One-way ANOVA analysis: ns = *P* > 0.05, “*” = *P* ≤ 0.05, “**” = *P* ≤ 0.01, “***” = *P* ≤ 0.001, “****” = *P* ≤ 0.0001. *n* = 5 independent experiments were performed for the aggregation of A30P aSyn in the presence and absence of CypA. One point of A30P aSyn aggregation was excluded from the analysis due to its low aggregation propensity and high deviation with respect to the mean value. Box plots represent the median (middle line), 25th, and 75th percentile (box), while the whiskers span from the minimum to the maximum value. **b** Residue-specific intensity changes observed in ^1^H–^15^N HSQC spectra of A30P aSyn upon addition of a fivefold excess of CypA. The CypA-induced intensity-broadening profile of wild-type aSyn is shown as a dashed line. **c**, ThT-fibrillization assay of the P128A-mutant aSyn protein alone (30 μM, blue) and in the presence of substoichiometric concentrations of CypA. Data points represent the mean values from *n* = 5 independent experiments ±1 SD.

The A30P mutation in aSyn causes an aggressive and early onset form of Parkinson’s disease^36^. The mechanistic basis of the increased pathogenic nature of the A30P mutation has not been resolved but might be connected to attenuated binding to vesicles^37^ and/or promotion of aSyn oligomerization^38^. In agreement with previous studies^38^, the A30P mutation, which introduces an additional proline residue, delayed fibrillization when compared to the wild-type protein (Fig. 1c,e), resulting in a ThT lag phase of 7–8 days (Fig. 4a). Nevertheless, the addition of CypA drastically shortened the lag phase of aSyn(A30P) to ~3 days (Fig. 4a). The enzyme also increased the speed of aSyn(A30P) fibril elongation, which is encoded in the slope of the ThT curve (Fig. 4a, right panel). NMR titrations with CypA further showed that the enzyme acts on the proline residue at position 30 in the mutant protein (Fig. 4b).

To further support the importance of *cis*-/*trans*-isomerization for aSyn aggregation, we replaced the proline at position 128 with alanine. The mutant protein aSyn(P128A), which only has four proline residues, formed fibrils more rapidly when compared to wild-type aSyn (Fig. 4c and Fig. 1c, e). The addition of substoichiometric amounts of CypA further decreased the lag phase by roughly a factor of two (Fig. 4c). The studies demonstrate that removal of a proline residue speeds up, while insertion of a proline residue through the Parkinson’s disease-associated A30P mutation delays fibrillization of aSyn. In both cases, however, CypA strongly accelerates fibrillization.

### CypA inhibits fibrillization of C-terminally truncated aSyn

**Fig. 5:**
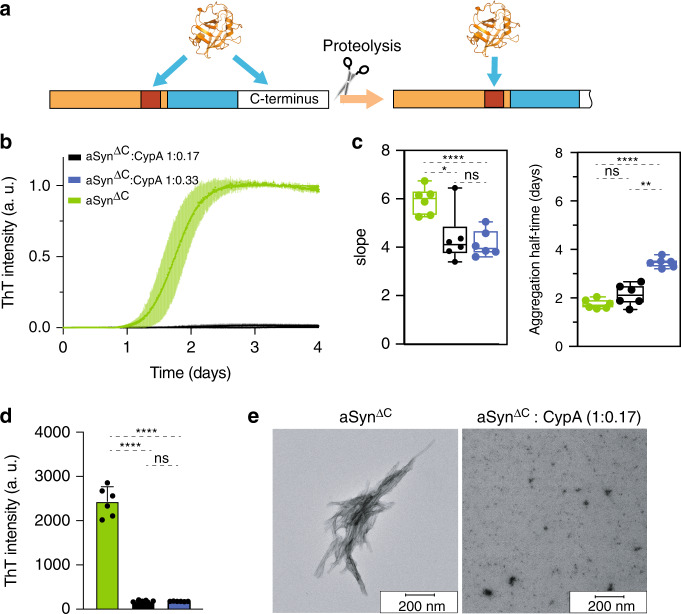
CypA blocks misfolding of C-terminally truncated aSyn. **a** Schematic representation of post-translational truncation of the C-terminal domain of aSyn. Truncation leads to a partial or full loss of the proline-rich region and exclusive binding of CypA to aSyn’s PreNAC. **b** ThT-fibrillization assay of C-terminally truncated aSyn alone (aSyn^ΔC^, 15 μM, green) and in the presence of substoichiometric concentrations of CypA. Error bars represent the standard deviation of *n* = 6 independent experiments. An independent experiment leading to the same results, can be found in Supplementary Fig. 8. **c** Aggregation kinetics of aSyn^ΔC^ in the absence (green) or presence of CypA (derived from ThT data in (**b**)). Box plots represent the median (middle line), 25th, and 75th percentile (box), while the whiskers span from the minimum to the maximum value. Note that the maximum ThT intensity is strongly decreased in the presence of CypA (see (**d**)). **d** Absolute values of ThT intensity after 4 days of aggregation of aSyn^ΔC^ in the absence (green) or presence of CypA. Data from aSyn^ΔC^:CypA molar ratios of 6:1 and 3:1 are shown in black and blue, respectively. Data points represent the mean values from *n* = 6 independent experiments ±1 SD. One-way ANOVA analysis: ns = *P* > 0.05, “*” = *P* ≤ 0.05, “**” = *P* ≤ 0.01, “***” = *P* ≤ 0.001, “****” = *P* ≤ 0.0001. **e** Representative electron micrographs of aSyn^ΔC^ samples at the end of the aggregation assay shown in (**b**). Ten electron micrographs were acquired.

Truncated versions of aSyn are present in the brains of Parkinson’s disease patients and include species terminating at residues 103, 115, 119, and 120 (Fig. 5a)^20,39^. The truncated aSyn proteins contain 0, 1, 2, and 3 prolines, respectively. As a model system for truncated aSyn, which lacks the proline-rich region, we prepared aSyn^ΔC^ stopping at A107. NMR spectroscopy showed that the truncation does not perturb the local structure of most of the 100 N-terminal residues of aSyn (Supplementary Fig. 7).

aSyn^ΔC^ does not contain any proline residues. In agreement with the absence of a proline *cis*-/*trans*- isomerization barrier, aSyn^ΔC^ aggregated more rapidly than both the wild-type protein and the P128A mutant aSyn (Fig. 5b, c)^40,41^. Strikingly, upon addition of only a small amount of CypA (aSyn:CypA molar ratio of 0:0.17), the ThT intensity was decreased by more than 90% (Fig. 5b, d and Supplementary Fig. 8). Electron microscopy supported the potent aggregation-inhibitory activity of CypA toward aSyn^ΔC^ (Fig. 5e). In addition, the kinetics of the residual aggregation of aSyn^ΔC^ decreased with increasing CypA concentration (Fig. 5c, right panel), in agreement with more aSyn^ΔC^ molecules bound to CypA.

### CypA captures the aSyn filament interface

**Fig. 6:**
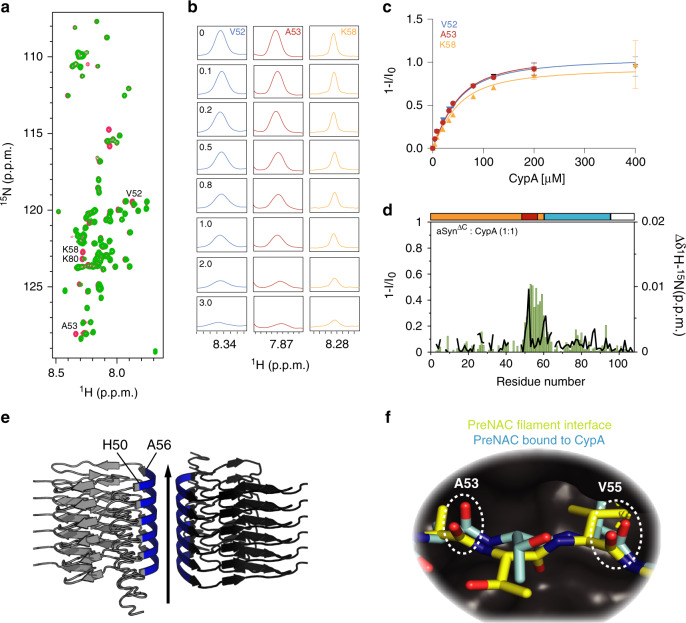
CypA captures aSyn residues important for filament-interface formation. **a** Superposition of ^1^H–^15^N HSQC spectra of aSyn^ΔC^ in the absence (red) and presence (green) of fivefold excess of CypA. **b** One-dimensional ^1^H traces through selected cross-peaks of ^1^H–^15^N HSQC spectra of aSyn^ΔC^ in the presence of increasing concentrations of CypA. CypA:aSyn^ΔC^ molar ratios are indicated in the panels of V52. **c** Intensity changes of residues in aSyn^ΔC^, which have predominantly slow-exchange behavior in the presence of CypA (see panel (**b**)). Error bars were derived from signal-to-noise ratios in the NMR spectra of each titration experiment. Lines represent global fits to the experimental data assuming a reversible 1-to-1 binding model. **d** Residue-specific intensity (1 − *I*/*I*_0_, green bars) and chemical shift changes (Δ*δ*_1H–15N_, black line) observed for cross-peaks of aSyn^ΔC^ in (**a**). The domain organization of aSyn^ΔC^ is shown on top**. e** The PreNAC region (blue) is part of the filament/filament interface in aSyn amyloid fibrils (PDB code: 6A6B, Supplementary Fig. 9). **f** Superposition of the filament structure of A53–V55 (PDB code: 6A6B, yellow) with the structure of a PreNAC peptide in complex with CypA (cyan, PDB code: 6I42).

To gain insight into the molecular basis of the aggregation-blocking chaperone activity of CypA toward aSyn^ΔC^ (Fig. 5), we performed NMR-interaction studies (Fig. 6a). Upon addition of an equimolar concentration of CypA, strong signal attenuation of the PreNAC residues in aSyn^ΔC^ was observed (Fig. 6a,b). Quantitative analysis of NMR signal attenuation in the PreNAC region for increasing CypA concentrations determined a *K*_D_ of 25.9 ± 3.1 μM (Fig. 6c). This *K*_D_ is below the *K*_D_ of the PreNAC region in wild-type aSyn for binding to CypA (*K*_D_ = 41 ± 6 μM)^31^. The increase in CypA affinity of the PreNAC region upon C-terminal truncation of aSyn indicates that the PreNAC and the C-terminal proline-rich region of aSyn compete for binding to the active site of CypA.

In the truncated aSyn^ΔC^ protein, the PreNAC region is the main CypA-interaction site (Fig. 6a, b). Comparison of the structure of the CypA/PreNAC complex^31^ with 3D structures of aSyn amyloid fibrils^14–17^ provides mechanistic insight into the strong aggregation-inhibitory activity of CypA toward aSyn^ΔC^ (Fig. 6d, e; Supplementary Fig. 9). In both assemblies, the PreNAC residues A53–V55 are involved in intermolecular contacts: in the CypA/PreNAC complex, they establish the interaction with CypA, while in aSyn amyloid fibrils, they are located in the interface between two filaments (Fig. 6d; Supplementary Fig. 9a). Notably, the atomic structure of residues A53–V55 in aSyn fibrils is similar to the conformation of these residues observed in complex with CypA (Fig. 6e and Supplementary Fig. 9b). The data suggest that CypA blocks the aggregation of C-terminally truncated aSyn through binding to residues that are required for the formation of the aSyn filament/filament interface.

### Conserved dual mechanism of PreNAC binding and isomerization catalysis

**Fig. 7:**
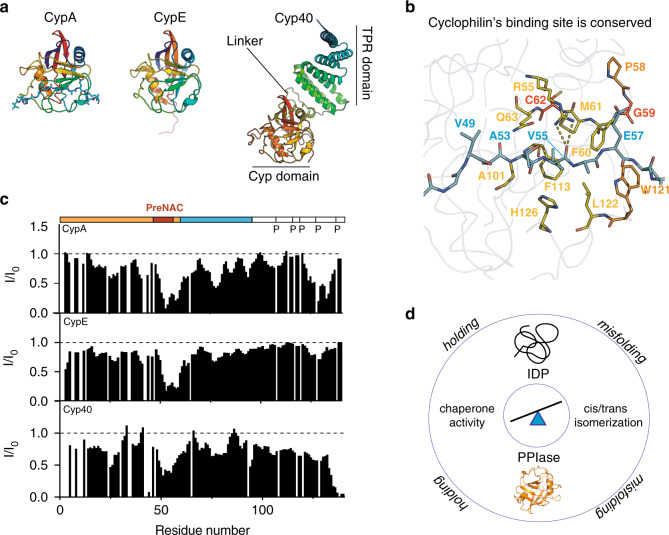
Conserved dual mechanism of *cis*-/*trans*-isomerization catalysis and molecular chaperone-like holding activity. **a** 3D structures of the three cyclophilins CypA (PDB code: 6I42), CypE (PDB code: 3UCH), and Cyp40 (PDB code: 1IIP). **b** Active site of CypA in complex with a peptide from the PreNAC region of aSyn (PDB code: 6I42). aSyn peptide residues labeled in blue. CypA residues of the active site are represented in yellow (conserved among CypA, CypE, and Cyp40), orange (residues mutated only in CypE or Cyp40), or red (residues not conserved among the three proteins). **c** Residue-specific intensity changes induced in aSyn in the presence of a 5-fold excess of CypA (top), CypE (middle), and Cyp40 (bottom). I_0_ and *I* are the intensities of ^1^H–^15^N HSQC cross-peaks in the absence and presence of enzymes, respectively. The domain organization of aSyn and the location of proline residues is shown on top. **d** Schematic representation of the tug-of-war between catalysis of proline isomerization and molecular chaperone activity: PPIase-catalyzed *cis*-/*trans*-isomerization lowers the energy barrier, which IDPs have to overcome during misfolding, while the chaperone-like holding activity of PPIases opposes aggregation.

To investigate if the binding to aSyn’s PreNAC region is relevant for other cyclophilins, we titrated aSyn with CypE and Cyp40 (Fig. 7a). The PPIase domains of CypA, CypE, and Cyp40 have high sequence similarity, including conserved residues in the active site (Fig. 7b and Supplementary Fig. 10). Several of these are involved in intermolecular contacts in the complex of CypA with an aSyn PreNAC peptide (Fig. 7b)^31^. In agreement with the high sequence conservation in their active site, all three cyclophilins caused broadening of ^1^H–^15^N cross-peaks belonging to aSyn’s PreNAC (Fig. 7c). In addition, broadening in proximity to the proline residues at aSyn’s C terminus, which bind independently of the PreNAC region to aSyn^31^, was present (Fig. 7c). Differences in the extent of cyclophilin-induced broadening point to different interaction modes of CypA, CypE, and Cyp40 with aSyn’s proline-rich region (Fig. 7c).

## Discussion

Catalysis of *cis*-/*trans*-isomerization of prolines is important for the cellular activity of proteins^42^. Because the isomerization of a prolyl peptide bond is slow, it is rate-limiting for the folding of proteins into a three-dimensional structure. PPIases, which catalyze *cis*-/*trans*-isomerization, help protein folding and thus belong to the class of molecular chaperones^4,5,42^. In contrast to globular proteins, IDPs do not have to undergo a folding process. The activity of molecular chaperones acting on IDPs is instead often related to the inhibition/reversal of disease-associated aggregation^43^.

Here, we show that the *cis*-/*trans*-isomerization of proline residues and molecular chaperone activity, i.e., inhibition of aggregation, by the PPIase CypA acting on the IDP aSyn is disconnected. Even more, the data show that catalysis of *cis*-/*trans*-isomerization and molecular chaperone activity is in a tug-of-war (Fig. 7d): catalysis of proline isomerization lowers the energy barriers, which aSyn has to overcome to aggregate into amyloid fibrils, while chaperone-like binding to a separate, disease-associated protein region, which does not contain proline residues, opposes aSyn aggregation. We further show that lowering the energy barriers for *cis*-/*trans*-isomerization in aSyn outpowers the aggregation-opposing holding activity of CypA (Fig. 1). However, CypA turns into a highly potent inhibitor of protein aggregation, when prolines and thus the need for catalysis are removed through post-translational truncation of aSyn (Fig. 5). The opposing effects are in agreement with findings that CypA has a PPIase-independent, general chaperone activity that is important for leukocyte chemotaxis^44^ and in vitro refolding of arginine kinase^45^. In addition, previous studies have shown opposing effects of CypA on protein folding at low and high CypA concentrations^46^.

Catalysis of *cis*-/*trans*-isomerization plays an important role in regulating the conformational ensemble of IDPs^47^. In addition, the structural promiscuity of IDPs makes them particularly suited to engage in protein–protein interactions^1^. Together with the high abundance of PPIases, this suggests an intimate link between IDPs and PPIases. The shallow architecture of the catalytic pocket of PPIase domains allows promiscuous binding of diverse sequences, independent of the presence of prolines^3^. Indeed, we observed binding to both the hydrophobic PreNAC and the proline-rich region of aSyn for three different cyclophilins (Figs. 3 and 7)^31^. The data are consistent with the multivalent nature of IDPs and their ability to engage in protein–protein interactions through multiple segments.

In the case of aSyn, the proline-rich region at the C terminus is separated from the aggregation-prone central domain. In other IDPs and disordered protein regions, amino-acid distributions might be more mixed such that aggregation-promoting isomerization activity and aggregation-inhibiting chaperone activity overlap. This will provide a further level of regulation to the activity and misfolding of IDPs through PPIases. Consistent with this hypothesis, Cyp40 disaggregates amyloid fibrils of the Alzheimer’s disease-associated protein tau, which contains proline residues in its aggregation-prone repeat domain^48^.

Our study reveals a tug-of-war between catalysis of *cis*-/*trans*-isomerization and molecular chaperone activity of PPIases and provides detailed insights into the enzymatic regulation of IDPs.

## Methods

### Recombinant expression and purification of proteins

The genes coding for human aSyn and its variants (A30P, P128, and aSyn^ΔC^) were cloned into a pT7-7 plasmid (Lansbury Laboratory, Harvard medical school, Cambridge, MA) and expressed in *Escherichia coli* BL21(DE3) cells (Novagen). Site-directed mutagenesis was carried out using a QuikChange kit from Qiagen, while polymerase chain reaction was conducted using the Phusion^®^ High-Fidelity DNA polymerase protocol (New England Biolabs, see Supplementary Table 1 for a complete primer list).

Transformed BL21(DE3) cells were grown at 37 °C until an absorbance *A*_600_ = 0.8 was reached and protein overexpression was induced with 0.5 mM IPTG at 25 °C o/n. Successively, cells were harvested and lysed by French press (Avestin EmulsiFlex-C3) in 20 mL of the following buffer: 10 mM Tris-HCl, pH 8.0, 1 mM EDTA, and 1 mM PMSF. The cell lysate was heated up to 98 °C for 20 minutes in a water bath. Cell debris and precipitated proteins were removed by centrifugation (30 min, 22,000*g*), and the supernatant was collected and incubated at 4 °C with streptomycin sulfate at a concentration of 10 mg/mL. The supernatant was collected by centrifugation (30 min, 22000*g*) and precipitated with ammonium sulfate added to the solution at a final concentration of 360 mg/mL, followed by incubation at 4 °C for 15 min. aSyn pellet was obtained and dialyzed against 25 mM Tris-HCl, 0.02% NaN_3_, pH 7.7 o/n. The dialysate was applied to an anion-exchange column (GE Healthcare Life Sciences, Mono Q 5/50 GL) and eluted by applying a salt gradient from 0 to 1 M NaCl. The aSyn fraction was eluted ~300 mM NaCl. Successively, the protein solution was concentrated and applied to a gel-filtration column (Superdex 75, GE Healthcare Life Sciences) and the buffer was exchanged with the following buffer: 100 mM NaCl, 50 mM HEPES, and 0.02% NaN_3_, pH 7.4.

The preparation of aSyn^ΔC^ was achieved following the same protocol, with the difference that after ammonium sulfate precipitation, the protein was dialyzed o/n in a buffer containing 25 mM TRIS, 0.02% NaN_3_, pH 7.0. The dialysate was applied to an anion-exchange column (GE Healthcare Life Sciences, Mono S 5/50 GL) and eluted by applying a salt gradient from 0 to 1 M NaCl. Buffer exchange was achieved by gel filtration.

For the production of ^15^N-labeled aSyn and its variants, M9-minimal medium supplemented with ^15^NH_4_Cl (Cambridge isotope Laboratories) was used. Finally, proteins were concentrated and dialyzed against the NMR buffer containing 100 mM NaCl, 50 mM HEPES, and 0.02% NaN_3_, pH 7.4.

The aSyn^Ctail^ peptide (residue 118–131 of aSyn) was synthesized by solid-phase peptide synthesis and purified using an HPLC column (P18 Eurospher, 8 × 250 mm, 5 µm, 100 A) in a buffer containing 0.1% of TFA.

The genes of human CypA and Cyp40 were cloned into a modified pET28a vector (Addgene), encoding an N-terminal TEV-cleavage-recognition site and 6× His tag. The plasmid coding for CypA was successively used as a template for the production of the CypA mutant CypA^R55A/K82A^. CypA site-directed mutagenesis was carried out using a QuikChange kit from Qiagen (see Supplementary Table 1).

CypA and CypA^R55A/K82A^ plasmids were transformed in *E. coli* BL21(DE3) cells (Novagen) and protein overexpression was induced through the addition of 0.4 mM IPTG to the cellular media until an absorbance of *A*_600_ = 0.6 was reached. After 14 h at 16 °C, cells were collected and resuspended in the following buffer: 300 mM NaCl, 20 mM HEPES, 3 mM DTT, 5 mM imidazole, and 0.02% NaN_3_, pH 7.4. After sonication and centrifugation, the supernatant was loaded onto a 5-ml Ni-Sepharose His-Trap column (GE Healthcare Life Sciences) and CypA was eluted with the resuspension buffer supplemented with 300 mM imidazole. TEV cleavage was carried out for 16 h at room temperature. Subsequently, CypA was loaded for the second time onto the Ni-Sepharose His-trap column, concentrated, and applied to a gel-filtration column (Superdex 75, GE Healthcare Life Sciences) equilibrated in the following buffer: 150 mM NaCl, 20 mM HEPES, 5 mM DTT, and 0.02% NaN_3_, pH 7.4. For the production of ^15^N- and/or ^15^N/^13^C-labeled CypA, M9-minimal medium supplemented with ^15^NH_4_Cl and/or ^13^C glucose (Cambridge isotope Laboratories) was used. Finally, proteins were concentrated and dialyzed against the NMR buffer containing 100 mM NaCl, 50 mM HEPES, and 0.02% NaN_3_, pH 7.4.

Cyp40 overexpression was achieved through the addition of 0.5 mM IPTG to the cellular media until an absorbance of *A*_600_ = 0.8 was reached. After 14 h at 20 °C, cells were collected and resuspended in the following buffer: 500 mM NaCl, 20 mM phosphate, 5 mM DTT, 5 mM imidazole, and 0.02% NaN_3_, pH 7.2. Successively, the lysate was sonicated and clarified by centrifugation. The clear lysate was loaded onto a 5-ml Ni-Sepharose His-Trap column (GE Healthcare Life Sciences) and Cyp40 was eluted with the resuspension buffer supplemented with 300 mM imidazole. TEV cleavage was carried out for 16 hours at room temperature. Subsequently, Cyp40 was loaded again onto the Ni-Sepharose His-trap column, concentrated, and applied to a gel-filtration column (Superdex 75, GE Healthcare Life Sciences) equilibrated in the following buffer: 150 mM NaCl, 20 mM HEPES, 5 mM DTT, and 0.02% NaN_3_, pH 7.2. Finally, proteins were concentrated and dialyzed against the buffer containing 100 mM NaCl, 50 mM HEPES, and 0.02% NaN_3_, pH 7.4.

CypE was purchased from Sigma Aldrich and diluted in NMR buffer containing 100 mM NaCl, 50 mM HEPES, and 0.02% NaN_3_, pH 7.4.

### aSyn fibrillization

Prior to the start of the fibrillization assay, samples of aSyn and its variants (300 μM) were ultracentrifuged at 163,700*g* for 2 h at 4 °C to remove preaggregated aSyn. Subsequently, the aSyn concentration was determined by UV spectroscopy, followed by dilution of aSyn in 600 μL of NMR buffer to a final concentration of 30 μM supplemented with or without CypA (or CypA^R55A/K82A^). A volume of 100 μL was pipetted into the wells of a flat-bottom black 96-well plate. Each reaction mixture contained 100 μL of 30 μM aSyn solution in NMR buffer and 50 μM of ThT. The same procedure was followed for aSyn^ΔC^, but the final concentration used in the assay was 15 μM.

In order to test the effect of CypA enzymatic activity on aSyn fibrillization, the CypA-binding site was blocked with CSA. CSA was diluted into a buffer containing 100% ethanol and added to CypA in twofold excess. The sample was then incubated for 2 hours at 4 °C under shaking. Subsequently, the samples were centrifuged and filtered to remove precipitated CSA. The clarified protein solution used for the aggregation assay was finally added to aSyn (30 μM) at a final concentration of 1 μM CypA and 2 μM CSA. The ethanol concentration of the final sample was less than 1%.

The plates were sealed with a transparent microplate sealer. The samples were shaken at a speed of 54 rpm every 10 min for a total duration of 1 min in a plate reader (Tecan, Spark 20 M) at 27 °C. ThT fluorescence was continuously measured from the top at a wavelength of 482 nm (excitation at 446 nm).

All experiments were performed four times. The measurements were averaged in order to get the final graph and the standard deviation was calculated among the measurements. Raw data were imported from Tecan SPARKCONTROL software (v. 2.2), prepared, and analyzed with Microsoft Excel (v. 16.16.13). The data were fitted to the following sigmoidal model using Graphpad Prism 8 software (San Diego, CA) and MATLAB (v. 2016b 9.1.0.441655, The MathWorks, Inc., Natick, Massachusetts, United States)

1 $$y = y_{\rm{0}} + \frac{{(y_{{\mathrm{fin}}} - y_{\rm{0}})}}{{1 + e^{\frac{{(x_{\rm{0}} - x)}}{b}}}},$$

where *y* is the ThT intensity signal, *x* is the time, *x*_0_ is the half-time of aggregation, *y*_0_ the initial fluorescence, 1/*b* describes the slope of the curve at its midpoint, and *y*_fin_ − *y*_0_ describes the total increase in fluorescence.

One-way analysis of variance analysis of the data was carried out with the standard package of Graphpad Prism 8 and the data were compared using Tukey’s multiple-comparison test.

### Electron microscopy

After aggregation, the samples were ultracentrifuged at 163,700*g* for 2 h at 25 °C to collect the fibrils. The supernatant was discarded and the pellet was carefully resuspended in an NMR buffer. Subsequently, the samples were bound to a glow-discharged carbon foil-covered grid. Afterward, the samples were negatively stained with 1% uranyl acetate and evaluated using a CM 120 transmission electron microscope (FEI, Eindhoven, The Netherlands). Pictures were taken with a Tietz F416 CMOS camera (TVIPS, Gauting, Germany).

### Western blot analysis

HEK cells were cultured in DMEM with 10% fetal bovine serum (FBS) in 10-cm dishes. About 72 h before harvesting, cells were cotransfected with 3 µg of polyethyleneimine per 1 µg of DNA (20-µg total of either pCMV6 empty vector or pCMV6FLAG-CypA along with pCDNA3.1 wild-type aSyn plasmid). For Sarkosyl fractionation, cells were harvested in Hsiao TBS Buffer and prepared as soluble and Sarkosyl-insoluble fractions as previously described^49^. Samples were analyzed with 4–20% sodium dodecyl sulfate gels (BioRad). After transfer, the polyvinylidene fluoride membrane was soaked for 30 min in 4% PFA and then blocked and probed. Antibody dilutions for aSyn (Sigma, Cat# S5566, 1:250), mouse anti-GAPDH (Proteintech, Cat# 60004, 1:5000), and rat anti-FLAG (Sigma, Cat# SAB4200071, 1:1000) were used and membranes developed using an LAS-4000 mini imager (GE Healthcare).

### Immunohistochemistry

HT-22 cells were cultured on Poly-l-Lysine (Sigma) coated coverslips (Fisher Scientific) in a 6-well dish in DMEM with 10% FBS (VWR) and then transfected with FLAG-CypA and wild-type aSyn plasmids, as described above. After 48 h, the media was aspirated and cells were washed with phosphate-buffered saline (PBS). Cells were fixed with 4% paraformaldehyde (Sigma) at room temperature for 20 minutes and then washed twice with PBS. Cells were permeabilized with PBS + 0.1% Triton X-100 (Fisher) for 10 min at room temperature and then blocked with 10% goat serum in PBS for 1 h at room temperature. Cells were incubated in primary antibodies (rat anti-FLAG (Sigma, Cat# SAB4200071) and mouse anti-aSyn, both at 1:500) overnight at 4 °C. Cells were then washed three times with PBS + 0.05% Triton X-100 and incubated with secondary antibodies at room temperature for 1 h (Alexa Fluor 647 anti-rat (Invitrogen) and Alexa Fluor 488 anti-mouse (Invitrogen), both at 1:1000). Coverslips were then washed two times with PBS + 0.05% Triton X-100 and then with a wash of PBS + DAPI (Sigma, Cat# 32670) at 1:500. Coverslips were then mounted using Prolong Gold (Invitrogen) and cured for 48 h before imaging.

### Fluorescence microscopy

A Zeiss LSM 880 AxioObserver laser-scanning confocal microscope with a 63×/1.40 PLAN APO oil objective was used for the colocalization-representative image. Five 1-µM Z-stacked images were taken using an argon laser for aSyn (488) signal and red helium–neon laser for FLAG signal (647). Representative images were made using Fiji v. 1.52 h.

### NMR-based interaction studies

NMR experiments were performed at room temperature, unless differently specified on 600-, 700-, 800-, 900-, and 950-MHz Bruker NMR spectrometers equipped with cryogenic probes. Titration experiments were acquired at 15 °C. Spectra were processed using Topspin (Bruker) and NMRpipe^50^ and analyzed with ccpnmr Analysis 2.2.1^51^. The combined ^1^H/^15^N chemical shift perturbation was calculated according to (((*δ*_H_)^2^ + (*δ*_N_/5)^2^)/2)^1/2^.

Unless otherwise specified, ^15^N-labeled aSyn at a concentration of 40 μM was titrated with fivefold excess of unlabeled CypA, CypA^R55A/K82A^, CypE, and Cyp40. ^1^H–^15^N HSQC spectra were acquired before and after the addition of each enzyme, with 2048 increments in the direct dimension and 256 points in the indirect dimension. In total, 64 transients were acquired with a recycle delay of 1.2 s. Chemical shift perturbation and intensity decay were monitored. The ^1^H–^15^N HSQC of the free protein was used as reference (*I*_0_) for calculation of the *I*/*I*_0_ ratio.

Due to the slow-exchange behavior of many peaks during the titration, the ligand-dependent intensity decrease was followed. The data were fitted assuming a simple two-state exchange-binding model between the free and the bound form according to

2 $$\left( {1 - \frac{I}{{I_{\rm{0}}}}} \right) = I_{{\mathrm{max}}}\left[ {\frac{{P_{\rm{0}} + x + K_D - \sqrt {(P_{\rm{0}} + x + K_D)^2 - 4P_{\rm{0}}x} }}{{2P_{\rm{0}}}}} \right],$$

where *I* is the intensity variation upon ligand addition and *I*_0_ is the intensity of the unbound free state, used as a reference, *P*_0_ is the total protein concentration, *K*_D_ the dissociation constant, and *x* the ligand concentration. Raw data were prepared and analyzed using Microsoft Excel (Version 16.16.13). Data fitting was performed using Graphpad Prism 8 (San Diego, CA) and MATLAB (v. R2016b 9.1.0.441655, The MathWorks, Inc., Natick, Massachusetts, United States).

Errors were evaluated from signal-to-noise ratios of NMR spectra and propagated according to

3 $$\sigma _I = \frac{I}{{I_{\rm{0}}}}\sqrt {\left( {\frac{{\sigma _I}}{I}} \right)^2 + \left( {\frac{{\sigma _{I0}}}{{I_{\rm{0}}}}} \right)^2},$$

where *σ*_I_ and *σ*_*I*0_ are the standard deviations of the noise in the spectra recorded in the presence and absence of a ligand, respectively.

^15^N-labeled aSyn was titrated with a fivefold excess of the complex CypA:CSA to analyze the inhibitory effect of CSA on the binding of CypA to aSyn. Due to its low water solubility, CSA was first dissolved in a buffer containing 100% ethanol and added to a solution of 200 μM CypA in twofold molar excess. Precipitated CSA was removed by filtering the sample with 0.22-μm cut-off centrifugal filters (Ultrafree ^®^—MC). The binding of CypA to CSA was investigated by the addition of CSA into a solution of 269 μM of ^15^N-labeled CypA in tenfold molar excess. Precipitated CSA was removed and the ^1^H–^15^N HSQC spectrum of the complex CypA:CSA was compared with the ^1^H–^15^N HSQC spectrum of free CypA.

### Solid-state NMR spectroscopy

Magic-angle spinning NMR experiments were performed at ~20 °C on an 800-MHz narrow-bore Bruker Avance III NMR spectrometer equipped with a 1.3-mm HCN probe. The spinning frequency was 55 kHz, and the cooling gas was set to 260 K. Spectra were referenced to the water proton line at 4.8 ppm, and reported on the DSS scale for ^13^C, and relative to liquid ammonia for ^15^N. Two 3D spectra were recorded on ^15^N/^13^C-labeled CypA for each fibril sample, namely, a 3D cross-polarization-based (H)CANH spectrum (~30 h), and a 3D (H)CCH spectrum (~72 h) using cross-polarization for all magnetization transfers, except the ^13^C–^13^C mixing, for which 1.7 ms of radio-frequency-driven recoupling was applied.

### Structure calculation of the CypA/aSyn^Ctail^ complex

To derive a structural model of the CypA/aSyn^Ctail^ complex, we first determined the backbone assignment of the aSyn^Ctail^ peptide (residues 118–131 of aSyn). To this end, the lyophilized peptide was resuspended at a concentration of 1 mM in buffer containing 100 mM NaCl, 50 mM HEPES, 0.02% NaN_3_, and 5% D_2_O. Two-dimensional ^1^H–^1^H TOCSY, ^1^H–^1^H NOESY, and natural abundance ^1^H–^15^N HSQC experiments were recorded at 5 °C, using both 700- and 900-MHz Bruker Avance NEO NMR spectrometers equipped with triple-resonance cryoprobes. ^1^H–^1^H TOCSY and ^1^H–^1^H NOESY experiments were acquired with 32 transients and a recycle delay of 1.2 s. The NOESY spectrum was acquired with a mixing time of 250 ms, while the TOCSY spectrum was recorded with an isotropic mixing period of 80 ms. Two-dimensional spectra were collected acquiring 2048 data points in the direct dimension and 256 increments in the indirect dimension. A ^1^H–^15^N HSQC experiment of aSyn^Ctail^ was acquired with 2048 increments in the direct f2 dimension and 140 increments in the f1 dimension. In total, 512 transients were acquired with a recycle delay of 1 s. Spectra were processed using Topspin v. 4.0.6 (Bruker) and NMRpipe 8.9^50^ and analyzed with ccpnmr Analysis v. 2.4.2^51^.

Next, an ensemble of 20 peptide structures was calculated introducing the experimental chemical shifts and NOE data into CYANA 3.98^52^. The lowest-energy scoring model, with no violation constraints, was selected for subsequent docking calculations with CypA using the HADDOCK webserver (v. 2.2)^53^. The residues of CypA, which experienced the strongest chemical shift perturbation and/or intensity loss upon addition of a fivefold excess of the aSyn^Ctail^ peptide, were selected as active residues during HADDOCK docking. In addition, the residues of aSyn showing the strongest chemical shift perturbation upon CypA binding were set as active residues during the docking of aSyn^Ctail^ to CypA. In the initial stage of the calculations, the two interacting partners were treated as rigid bodies and a rigid-body energy-minimization step was performed. During this stage, 1000 structures were calculated. Subsequently, a total of 200 structures were selected and subjected to semiflexible refinement, followed by refinement in explicit water. The structures were divided into ten clusters and the most representative structure of the CypA/aSyn^Ctail^ complex was selected on the basis of the lowest HADDOCK score.

To further explore the conformational space in the bound state, we performed structure calculations with the Rosetta software starting from the lowest HADDOCK score structure. Initially, this structure was subjected to the pre-pack option within the Rosetta “FlexPepDock” application to remove structural clashes. Next, the “*ab-initio*” and the “*peptide refine*” options were selected during the production calculation (100,000 structures) using the “FlexPepDock” ab initio protocol^54^. Finally, five major clusters (with a maximum number of 20 models per cluster) were extracted with the “*energy_based_clustering*” program in the Rosetta suite.

### Reporting summary

Further information on experimental design is available in the Nature Research Reporting Summary linked to this paper.

## Supplementary information

Supplementary Information

Reporting Summary

## Data Availability

Source data are provided with this paper. Other data that support the findings of this study are available from the corresponding author upon reasonable request. Source data are provided with this paper.

## Source data

Source Data
