# Supplementary material for: Catalysis of proline isomerization and molecular chaperone activity in a tug-of-war
Source: Nat Commun. 2020 Nov 27;11:6046. doi: 10.1038/s41467-020-19844-0 (PMC7695863; doi:10.1038/s41467-020-19844-0)
Supplement: Supplementary file 1 — Supplementary Information [file 41467_2020_19844_MOESM1_ESM.pdf]

Supplementary Information for

Catalysis of proline isomerization and molecular chaperone activity in a tug-of-war

Filippo Favretto[1], David Flores[1], Jeremy D. Baker[2], Timo Strohäker[1], Loren B. Andreas[3], Laura J. Blair[2], Stefan Becker[3] and Markus Zweckstetter[1,3]

[1] German Center for Neurodegenerative Diseases (DZNE), Von-Siebold-Str. 3a, 37075 Göttingen, Germany.
[2] Department of Molecular Medicine, Morsani College of Medicine, USF Health Byrd Alzheimer's Institute, University of South Florida, Tampa, FL 33613, USA.
[3] Department for NMR-based Structural Biology, Max Planck Institute for Biophysical Chemistry, Am Faßberg 11, 37077 Göttingen, Germany.

Supplementary Figures

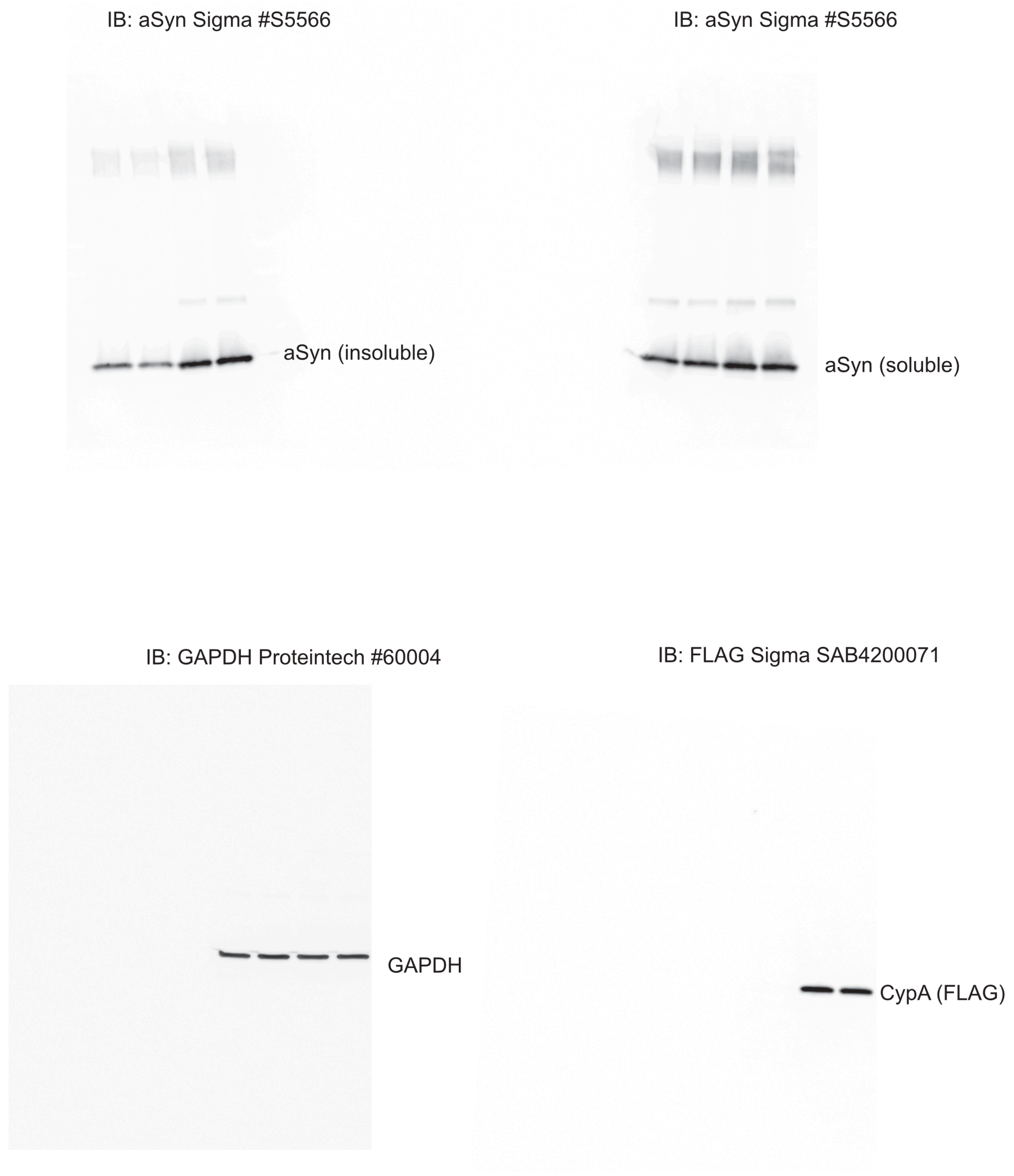

Supplementary Fig. 1 | Uncut blots from Fig. 1b, labelled with the antibody information.

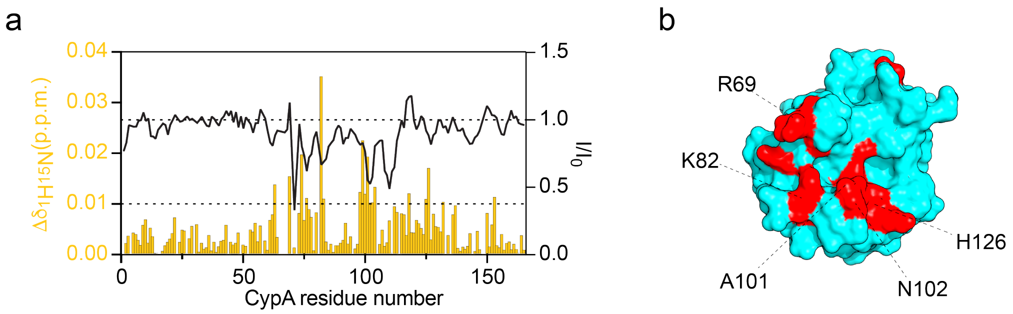

Supplementary Fig. 2 │ NMR spectroscopy of the binding of aSyn^{Ctail} (residue 118-131 of aSyn) to CypA. a, Residue-specific perturbation of $^{1}H/^{15}N$ chemical shifts (yellow bars) and cross-peak intensity changes (black line) in CypA upon addition of a five-fold excess of aSyn^{Ctail}. **b,** CypA residues with normalized $^{1}H/^{15}N$ chemical shift perturbations exceeding 0.01 (panel (a)) are marked in red in the 3D structure of CypA (PDB code: 6I42).

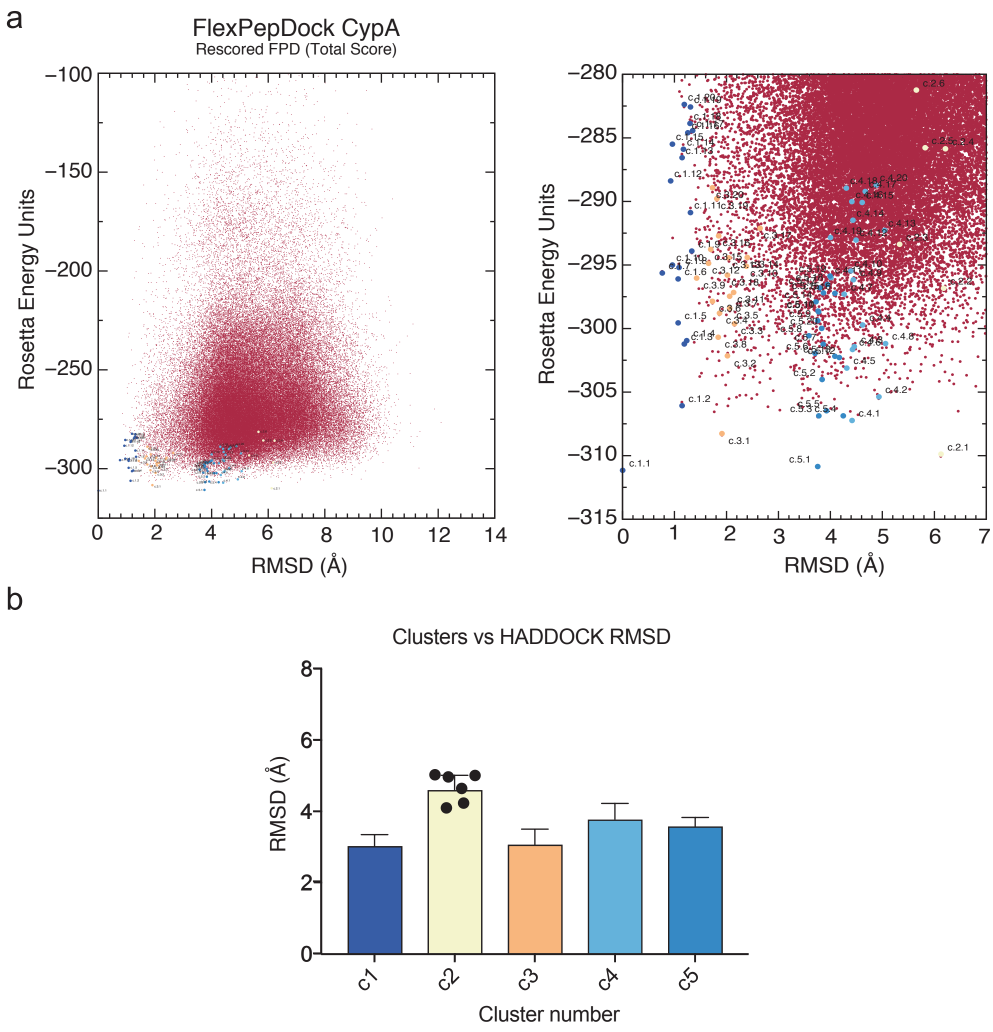

Supplementary Fig. 3 | Rosetta energy versus root-mean square deviation (RMSD) against the HADDOCK docking model of the CypA/aSyn^{Ctail}-complex. **a,** Full distribution for 100,000 Rosetta-calculated structures (left) with low energy/RMSD conformations shown to the right. **b,** RMSD values of the five Rosetta-derived lowest-energy clusters with respect to the HADDOCK docking model. Data points represent the mean values of the RMSD of the different structure in a specific cluster calculated from n = 20 structures +/- one SD. Only 6 structures were present in cluster 2.

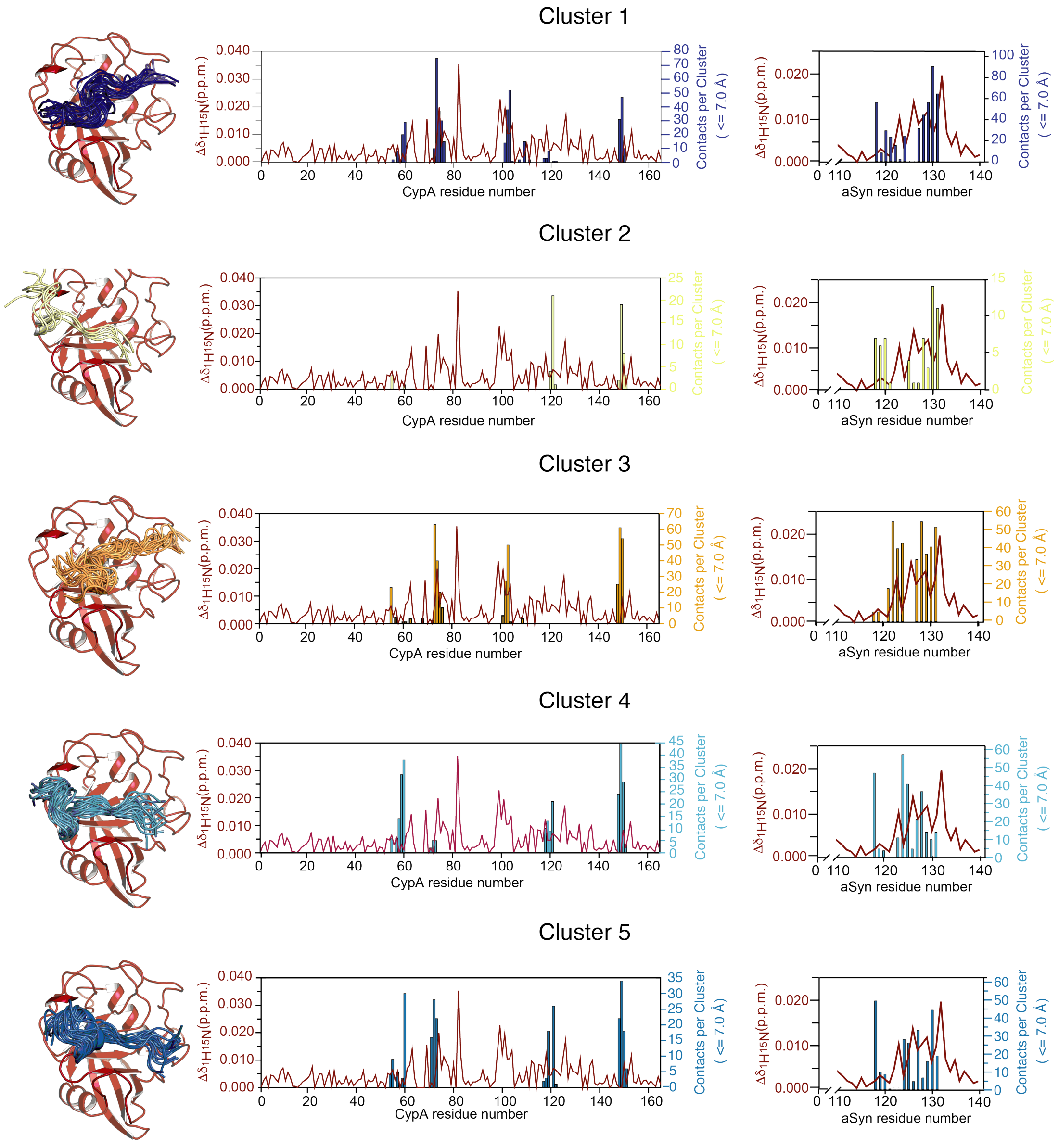

Supplementary Fig. 4 | Comparison of the five Rosetta-calculated lowest-energy clusters. For each cluster, the ensemble of 20 complex structures (left) is shown together with comparisons of $^{1}H/^{15}N$ chemical shift perturbations induced by $aSyn^{Ctail}$ in CypA (middle; also shown in Supplementary Fig. 1a), as well as by CypA in the C-terminal domain of full-length aSyn (right), with the number of intermolecular contacts between CypA and $aSyn^{Ctail}$ in each cluster (bars).

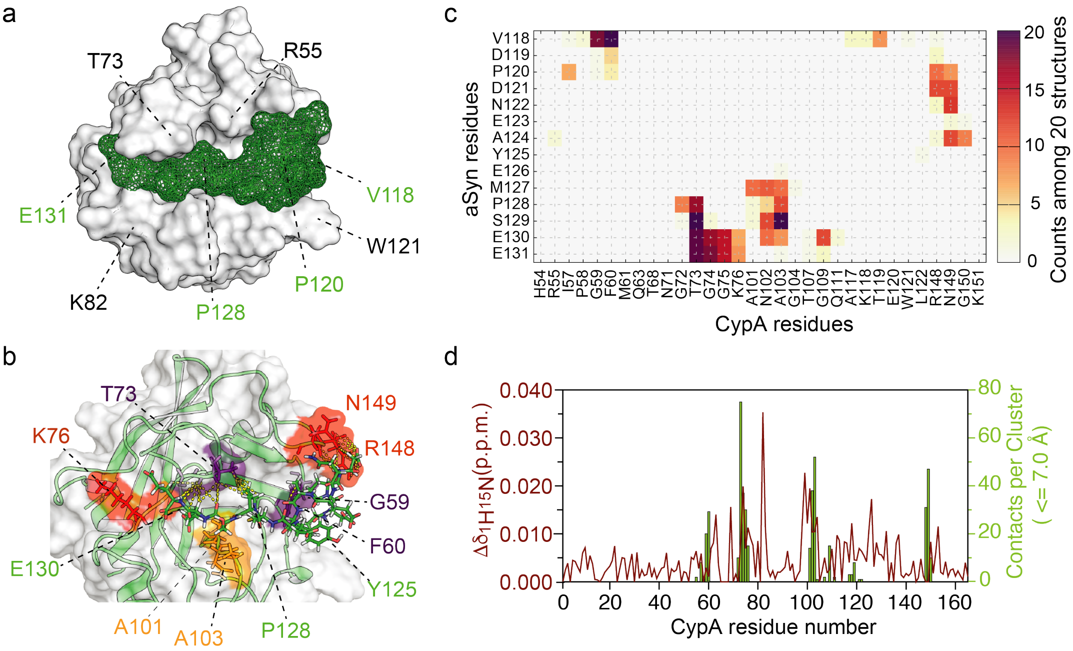

Supplementary Fig. 5 | Structure of the aSyn proline-rich region bound to CypA. **a,** Lowest-energy conformer of the $CypA/aSyn^{Ctail}$-complex derived from Rosetta calculations. $aSyn^{Ctail}$ in green, CypA in grey (CypA residues in black). **b,** Zoomed view of the hydrophobic pocket of CypA in the $CypA/aSyn^{Ctail}$-complex. CypA residues with multiple intermolecular contacts are highlighted (color code as in (c)). aSyn residues in green. **c,** Heat map of intermolecular contacts in cluster 1 of the Rosetta-derived $CypA/aSyn^{Ctail}$-complex. **d,** Comparison of aSyn-induced ^{1}H-^{15}N chemical shift perturbation in CypA (line) with the residue-specific number of contacts from $aSyn^{Ctail}$ to CypA (green bars) in cluster 1 of the Rosetta-derived $CypA/aSyn^{Ctail}$-complex.

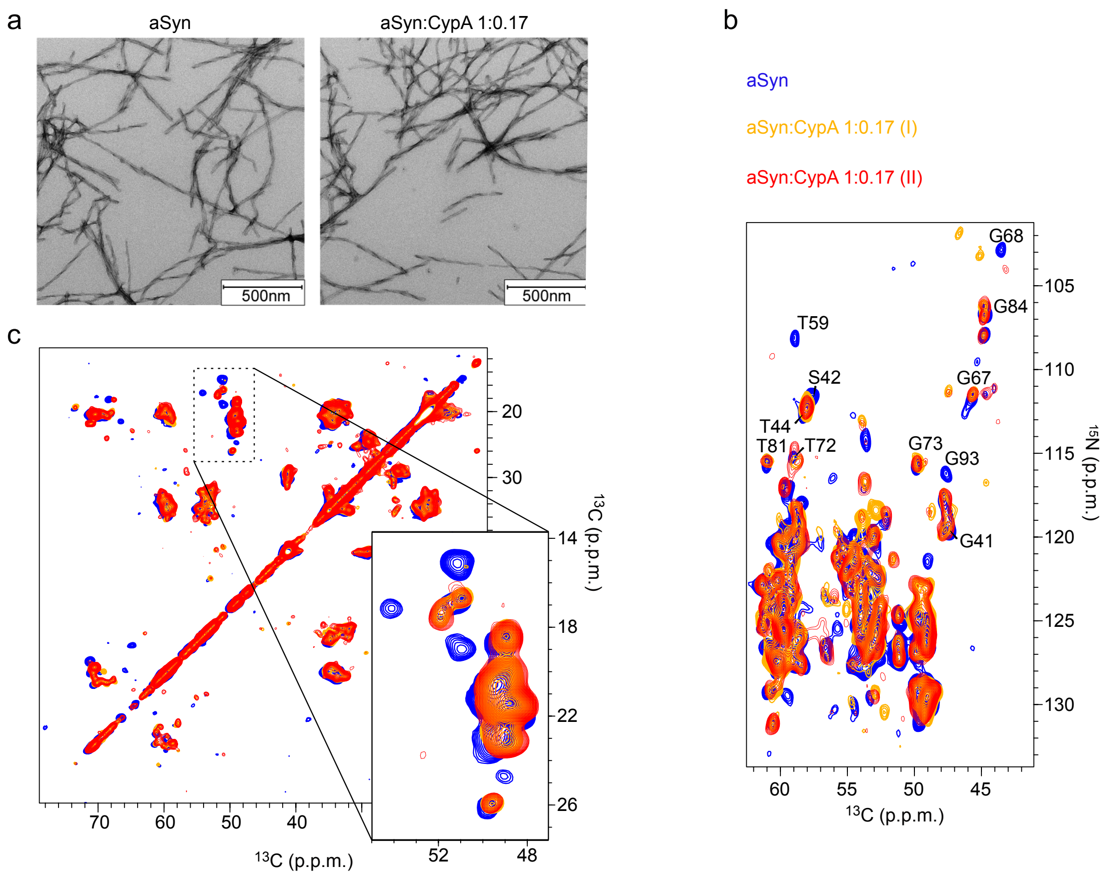

Supplementary Fig. 6 | CypA activity changes aSyn amyloid structure. a, Representative electron micrographs of aSyn fibrils formed in the absence (left) and presence of a substoichiometric concentration of CypA (right; aSyn:CypA molar 1:0.17). Ten micrographs were taken for each condition. **b-c,** Superposition of the N-Ca projection of a (H)CANH spectrum (b) and C-C projection of an (H)CCH spectrum (c) of aSyn fibrils formed in the absence (blue) and presence (orange) of a substoichiometric concentration of CypA; aSyn:CypA 1:0.17 by mole. Comparison with the spectra of a third sample of aSyn fibrils (red), which was independently aggregated in presence of CypA, showed similar but not identical changes. This indicates that some of the spectral changes are due to intrinsic sample variations, while the majority of changes is caused by the activity of CypA.

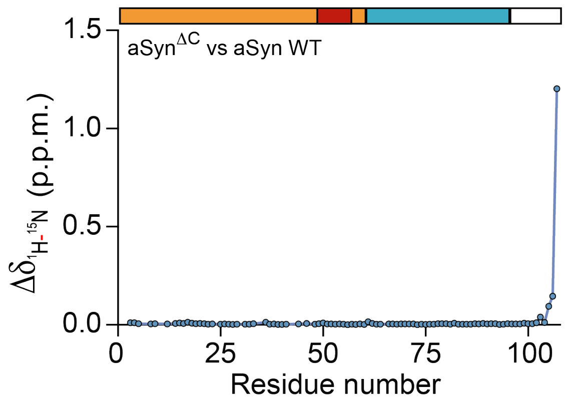

Supplementary Fig. 7 │ aSyn and aSyn$^{\Delta C}$ have similar local structural properties. Normalized averaged ^{1}H-^{15}N chemical shift differences between aSyn and the C-terminally truncated protein aSyn$^{\Delta C}$, which stops at A107.

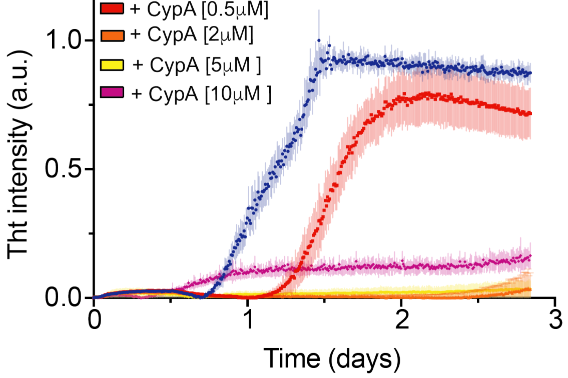

Supplementary Fig. 8 │ Influence of low CypA concentrations on the fibrillization of C-terminally truncated aSyn. Data points and error bars represent average values and standard deviation from n=4 independent experiments.

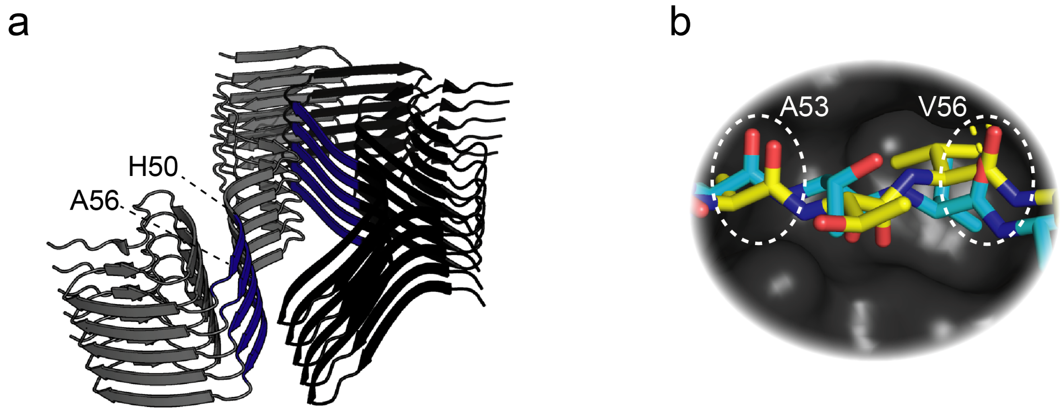

Supplementary Fig. 9 | Comparison of the conformation of residues A53-V55 of aSyn in complex with CypA and in aSyn fibrils extracted from a MSA patient. a, Structure of aSyn fibrils extracted from a MSA patient (PDB code: 6XYO). Residues H50-A56 are highlighted in blue. **b,** Superposition of the MSA filament structure of A53-V55 (PDB code: 6XYO; yellow) with the structure of a PreNAC peptide in complex with CypA (cyan; PDB code: 6I42).

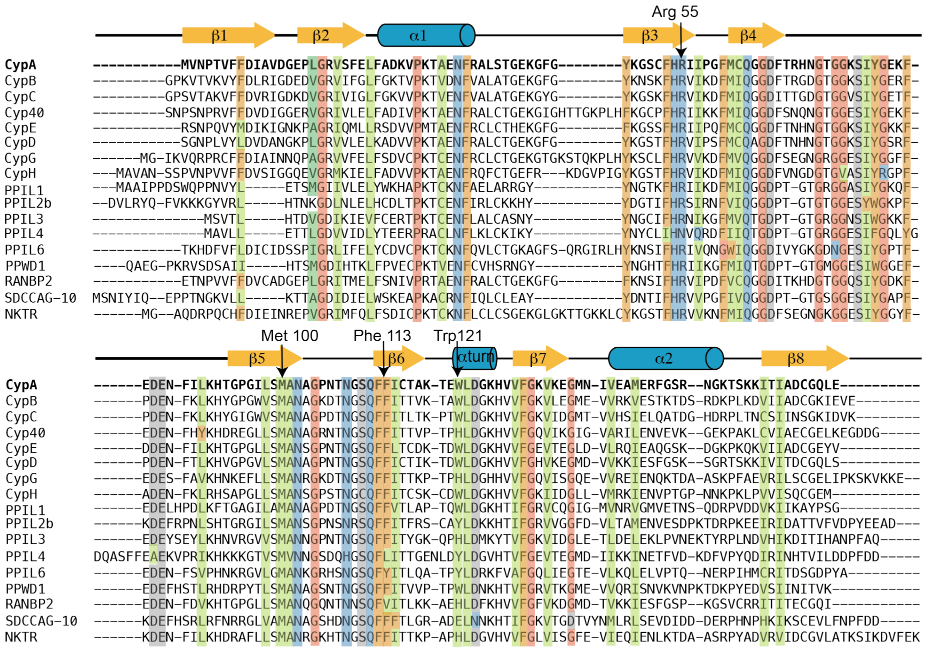

Supplementary Fig. 10 | Alignment of 17 human cyclophilin sequences. Conserved secondary structure elements and functionally important residues are shown on top.

Primer name	Variant	Primer sequence
CypA_R55A_fw 5’-3’	CypA^{R55A}	GTTCCTGCTTTCACGCAATTATTCC
CypA_R55A_rv 5’-3’	CypA^{R55A}	GGAATAATTGCGTGAAAGCAGGAAC
CypA_K82A_fw 5’-3’	CypA^{K82A}	CTATGGGGAGGCATTTGAAGATGAG
CypA_K82A_rv 5’-3’	CypA^{K82A}	CTCATCTTCAAATGCCTCCCCATAG
aSyn_P128A_fw 5’-3	aSyn^{P128A}	GCTTATGAAATGGCTTCTGAGGAAGG
aSyn_P128A_rv 5’-3’	aSyn^{P128A}	CCTTCCTCAGAAGCCATTTCATAAGC
aSyn_107-Stop_fw 5’-3’	aSyn$^{\Delta C}$	GAATGAAGAAGGAGCCTAACAGGAAG GAATTCTGG
aSyn_107-Stop_rv 5’-3’	aSyn$^{\Delta C}$	CCAGAATTCCTTCCTGTTAGGCTCCT TCTTCATTC

Supplementary Table 1 │ List of primers used in this work.
