# Supplementary material for: Catalysis of proline isomerization and molecular chaperone activity in a tug-of-war
Source: Nat Commun. 2020 Nov 27;11:6046. doi: 10.1038/s41467-020-19844-0 (PMC7695863; doi:10.1038/s41467-020-19844-0)
Supplement: Supplementary file 2 — Reporting Summary [file 41467_2020_19844_MOESM2_ESM.pdf]

nature research

Corresponding author(s): Markus Zweckstetter

Last updated by author(s): Oct 14, 2020

Reporting Summary

Nature Research wishes to improve the reproducibility of the work that we publish. This form provides structure for consistency and transparency in reporting. For further information on Nature Research policies, see Authors & Referees and the Editorial Policy Checklist .

Statistics

For all statistical analyses, confirm that the following items are present in the figure legend, table legend, main text, or Methods section.

n/a	Confirmed	
☐	☒	The exact sample size (*n*) for each experimental group/condition, given as a discrete number and unit of measurement
☐	☒	A statement on whether measurements were taken from distinct samples or whether the same sample was measured repeatedly
☐	☒	The statistical test(s) used AND whether they are one- or two-sided<br>*Only common tests should be described solely by name; describe more complex techniques in the Methods section.*
☐	☒	A description of all covariates tested
☒	☐	A description of any assumptions or corrections, such as tests of normality and adjustment for multiple comparisons
☐	☒	A full description of the statistical parameters including central tendency (e.g. means) or other basic estimates (e.g. regression coefficient) AND variation (e.g. standard deviation) or associated estimates of uncertainty (e.g. confidence intervals)
☐	☒	For null hypothesis testing, the test statistic (e.g. *F*, *t*, *r*) with confidence intervals, effect sizes, degrees of freedom and *P* value noted<br>*Give P values as exact values whenever suitable.*
☒	☐	For Bayesian analysis, information on the choice of priors and Markov chain Monte Carlo settings
☒	☐	For hierarchical and complex designs, identification of the appropriate level for tests and full reporting of outcomes
☒	☐	Estimates of effect sizes (e.g. Cohen's *d*, Pearson's *r*), indicating how they were calculated

Our web collection on statistics for biologists contains articles on many of the points above.

Software and code

Policy information about availability of computer code

Data collection	Bruker NMR (600, 700, 800, 900, 950 MHz) and Bruker TopSpin v 4.0.6, Tecan SPARKCONTROL software v 2.2, Tietz F416 CMOS camera. Zeiss LSM 880 AxioObserver laser scanning confocal microscope was used to acquire fluorescence microscopy images.
Data analysis	Bruker TopSpin v 4.0.6, CcpNmr Analysis v 2.4.2, NMRPipe v 8.9, MTLAB R2016b (9.1.0.441655), Graphpad Prism 8, Microsoft Excel (Version 16.16.13), Adobe illustrator CS51 v 15.1.0 was used for producing final high quality images. CYANA 3.98 was used to prepare the peptide ensemble. HADDOCK 2.2 was used to prepare the CypA/peptide complex. Rosetta 2.x ("FlexPepDoc" ab initio protocol) was used to get our molecular model (CypA:aSyn 118-131). The software Fiji v 1.52h was used to prepare representative fluorescence images. ChemDraw v 19.1 was used to draw the structure of Cyclosporin A.

For manuscripts utilizing custom algorithms or software that are central to the research but not yet described in published literature, software must be made available to editors/reviewers. We strongly encourage code deposition in a community repository (e.g. GitHub). See the Nature Research guidelines for submitting code & software for further information.

Data

Policy information about availability of data

All manuscripts must include a data availability statement. This statement should provide the following information, where applicable:

- Accession codes, unique identifiers, or web links for publicly available datasets
- A list of figures that have associated raw data
- A description of any restrictions on data availability

All the PDB codes cited in this article (PDB: 6A6B, PDB: 6I42, PDB: 3UCH, PDB: 1IIP, PDB: 6XYO) are available from the protein data bank web server.
Row data are available for Figures 6c, 5b-c-d and 2c as an Excel File (Data Availability).
Data that support the findings of this study are available from the corresponding authors on reasonable request.

Field-specific reporting

Please select the one below that is the best fit for your research. If you are not sure, read the appropriate sections before making your selection.

☒ Life sciences ☐ Behavioural & social sciences ☐ Ecological, evolutionary & environmental sciences

For a reference copy of the document with all sections, see nature.com/documents/nr-reporting-summary-flat.pdf

Life sciences study design

All studies must disclose on these points even when the disclosure is negative.

Sample size	Aggregation assays for aSynWT in presence and absence of CypA/CypAR55A-K82A/CypA-CsA, were repeated 4 times in parallel (n = 4 independent experiments) . Aggregation assays for aSynA30P and aSynP128A in presence and absence of CypA were repeated 5 times in parallel (n = 5 independent experiments). Aggregation assays for aSynDC (C-terminal truncated) were repeated 6 times (n = 6 independent experiments). In this way we ensured to have an amount of data points which was statistically relevant (at least 3 repetitions per each experimental condition). These data were used to calculate average and standard deviation. Cell imaging assays in HT-22 cells were repeated in 2 times (n = 2 independent experiments) using at least 10 fields of view from each condition. HEK293T cell experiments were repeated at least 3 times (N=3 independent experiments).
Data exclusions	Data that were varying more than 3 standard deviations with respect to the average or that were giving a significant reduction of Tht intensity at the final aggregation point were excluded from the analysis. With these criteria we excluded a sample from the analysis of the aggregation of aSynA30P.
Replication	All our experiments were reproducible.
Randomization	For each cell culture experiment, the total number of cells from each cell line required for a single experiment was pooled and seeded randomly into plates for the experiments where they were treated.
Blinding	For cell imaging experiment, imaging areas were selected based off location of aSyn injected cells, so the user was blinded to CypA levels in these areas before imaging.

Reporting for specific materials, systems and methods

We require information from authors about some types of materials, experimental systems and methods used in many studies. Here, indicate whether each material, system or method listed is relevant to your study. If you are not sure if a list item applies to your research, read the appropriate section before selecting a response.

Materials & experimental systems

n/a	Involved in the study	
☐	☒	Antibodies
☐	☒	Eukaryotic cell lines
☒	☐	Palaeontology
☒	☐	Animals and other organisms
☒	☐	Human research participants
☒	☐	Clinical data

Methods

n/a	Involved in the study	
☒	☐	ChIP-seq
☒	☐	Flow cytometry
☒	☐	MRI-based neuroimaging

Antibodies

Antibodies used	Anti-FLAG, Rat monoclonal antibody (Sigma, Cat# SAB4200071); Anti-aSynuclein antibody, Mouse monoclonal (Sigma, Cat# S5566); anti-GAPDH, mouse monoclonal antibody (Proteintech, Cat# 60004). Secondary antibodies: Alexa Fluor 647 anti-rat (Invitrogen, Cat# A-21247). Alexa Fluor 488 anti-mouse (Invitrogen, Cat# A-11001).
Validation	FLAG antibody - There are 19 citations of this antibody on the Sigma website and was validated by the manufacturer for IP and WB. Validation is also confirmed in our Western where bands show in the CyPA-FLAG transfected samples but not in the empty vector control samples.<br>aSynuclein antibody - There are 18 citations of this antibody on the Sigma website and was validated by the manufacturer for IP, IHC, and WB. The manufacturer shows positive bands for recombinant aSynuclein on a Western blot.<br>GAPDH antibody - There are 1696 citations of this antibody on the Proteintech website with 18 validation examples. It has been validated for WB, IP, IHC, IF, FC, and ELISA. PyMol v 1.7.6.0 was used to prepare the molecular structures of the proteins studied in this work.<br>anti-rat 647 - There are 60 citations of this antibody on the ThermoFisher Scientific website and was validated by the manufacturer for ICC, IF, IHC, IP and WB.<br>anti-mouse 488 - There are 938 citations of this antibody on the ThermoFisher Scientific website and was validated by the

manufacturer for Flow, ICC, IF, and IHC.

Eukaryotic cell lines

Policy information about cell lines

Cell line source(s)	HT22 cells (direct from Salk Institute, where this reagent was generated in January 2017); HEK293T cells (purchased from ATCC January 2016) Lot 62278039
Authentication	Cell lines were authenticated when they were supplied from Salk and ATCC, respectively HEK293T cells were tested by ATCC for ampule passage number, population doubling level, total cells, post-freeze viability, growth phase properties, morphology, mycoplasma, species determination, sterility and human pathogenic virus testing. HT22 cells were tested for morphology, PCR, Northern blot and glutamate sensitivity analysis.
Mycoplasma contamination	Cell lines were not tested for mycoplasm contamination
Commonly misidentified lines (See ICLAC register)	No cells in our study are listed in ICLAC.
